# Female Sexual Function and Pelvic Floor Muscle Training: A Narrative Review

**DOI:** 10.7759/cureus.85751

**Published:** 2025-06-11

**Authors:** Dimitrios Stamos, Vaia Sapouna, Katerina Maria Astraka, Sofia Thanopoulou, Ioannis Giannakis, Agni Pantou, Dimitrios Baltogiannis, Minas Paschopoulos, Nikolaos Sofikitis, Athanasios Zachariou

**Affiliations:** 1 Department of Urology, University of Ioannina, Ioannina, GRC; 2 Clinical Exercise Physiology and Rehabilitation Laboratory, Department of Physiotherapy, University of Thessaly, Lamia, GRC; 3 Department of Urology, Outpatient Clinic, KENTAVROS Physical Medicine and Rehabilitation Center, Volos, GRC; 4 Department of Obstetrics and Gynecology, University of Ioannina, Ioannina, GRC

**Keywords:** female, pelvic floor disorders, pelvic floor muscle training, physical therapy, sexual function

## Abstract

Female sexual dysfunction (FSD) is a multifactorial condition affecting desire, arousal, orgasm, and satisfaction, with wide-ranging implications for women's physical and emotional well-being. Although prevalent, especially among postmenopausal and postpartum populations, FSD remains under-recognized and undertreated. Pelvic floor muscle training (PFMT) has emerged as a promising, non-invasive therapeutic approach for managing FSD, particularly when associated with pelvic floor disorders such as urinary incontinence, pelvic organ prolapse, and overactive bladder. This narrative review synthesizes anatomical, physiological, clinical, and therapeutic insights into the relationship between pelvic floor function and female sexual health. The pelvic floor’s structural complexity-comprising muscular, connective, and neurovascular elements-plays a crucial role in sexual response. Dysfunction of this system can contribute to sexual pain, reduced arousal, and orgasmic disorders. PFMT, involving voluntary muscle contractions, biofeedback, or electrical stimulation, has demonstrated benefits across diverse female populations. Variables such as frequency, intensity, supervision, and duration of PFMT significantly influence its effectiveness. Evidence suggests that PFMT improves sexual function in general populations and is particularly beneficial for postpartum, postmenopausal women, and those with neurological or gynecological issues. Improvements are seen in sexual desire, arousal, lubrication, orgasm, and pain reduction. The mechanisms underlying these effects include enhanced muscle strength, increased genital blood flow, and psychological improvements such as body awareness and reduced anxiety. Despite strong supportive evidence, implementation challenges persist, including adherence difficulties, a lack of standardized protocols, and insufficient professional training. Barriers to adherence include misconceptions, discomfort, lack of motivation, and poor understanding of proper technique. Facilitators include clear guidance, customized approaches, technological tools, and professional supervision. Mobile health applications and patient empowerment strategies show promise in enhancing engagement and outcomes. Future research should focus on long-term efficacy, standard intervention protocols, and the integration of PFMT with other therapies, such as pharmacological treatments. Overall, PFMT represents a low-risk, cost-effective intervention capable of significantly improving quality of life and sexual function in women across the lifespan.

## Introduction and background

Sexual relationships are fundamental to human reproduction and play a key role in the overall quality of life. Despite increasing recognition of sexual health as an essential component of general well-being, female sexual dysfunction (FSD) remains frequently overlooked. This under-recognition can have far-reaching consequences for women’s physical, psychological, and emotional health. The female sexual response cycle is typically divided into interrelated phases: desire, arousal, orgasm, and resolution [1]. However, research by Rosemary Basson and colleagues has highlighted that women’s sexual response often follows a nonlinear pattern in which emotional intimacy, contextual factors, and subjective arousal play central roles [2]. Often, sexual desire may emerge during or after arousal rather than preceding it, challenging the traditional linear models of sexual function. Sexual functioning itself is a complex process involving neurological, vascular, and hormonal regulation.

FSD refers to persistent or recurrent problems in one or more phases of the sexual response cycle that interfere with a woman’s sexual satisfaction and functioning. It encompasses a range of psychological and physiological issues, including diminished sexual desire, difficulty with arousal or orgasm, and disorders associated with sexual pain, such as dyspareunia and vaginismus [3]. These conditions can profoundly affect a woman’s intimate relationships and quality of life. While FSD can occur at any age, its prevalence often increases with age due to hormonal shifts related to menopause [3].

Epidemiological studies indicate that FSD is a highly prevalent issue globally. A recent meta-analysis estimated the pooled prevalence among women of reproductive age to be 50.75% (41.73-59.78). Specific types of dysfunctions were reported at varying rates: pain (39.08%), desire disorders (50.70%), arousal issues (48.21%), lubrication difficulties (37.60%), orgasmic problems (40.16%), and dissatisfaction (35.02%) [4]. The highest prevalence is often observed in perimenopausal women aged 51-59, where contributing factors may include lower educational attainment, relationship stress, and comorbid conditions such as diabetes, hypertension, or chronic vulvar inflammation [5]. Both surgical and natural menopause have been associated with increased risks of sexual dysfunction [6,7].

In addition, pelvic floor disorders are a significant contributor to sexual dysfunction in women. Conditions such as urinary incontinence, pelvic organ prolapse, fecal incontinence, and pelvic-perineal pain can disrupt sexual activity by causing discomfort, embarrassment, or fear of intimacy [8,9]. Notably, a positive association has been identified between pelvic floor muscle strength (PFMS) and sexual function, particularly in women experiencing stress urinary incontinence [10]. The pelvic floor muscles, located at the base of the pelvis, are essential for maintaining continence, supporting pelvic organs, and enabling pleasurable and pain-free sexual activity [11]. Strengthening these muscles through pelvic floor exercises is both accessible and effective, particularly in women experiencing pelvic or genital pain [1].

Given the widespread prevalence and consequences of FSD and the key role pelvic floor function plays in female sexual health, a thorough evaluation of pelvic floor muscle training (PFMT) as a therapeutic approach is warranted. This review aims to synthesize existing evidence on the impact of PFMT on female sexual function across diverse populations, offering insights into its mechanisms and clinical potential. Through this analysis, we seek to highlight the broader implications of PFMT in promoting women's sexual health and enhancing overall well-being.

## Review

Anatomy and physiology of the female pelvic floor 

The term *pelvic floor* refers to the complex musculoskeletal structure that seals the inferior boundary of the pelvic cavity. It comprises the pelvic floor muscles (PFMs), ligaments, connective tissue (fascia), blood vessels, nerves, and pelvic organs such as the bladder, uterus, and rectum. This dome-shaped system is critical not only for supporting pelvic viscera but also for maintaining continence, contributing to sexual function, facilitating childbirth, and stabilizing the trunk during movement [12-14].

Anatomically, the pelvic floor is divided into three main muscle layers. The superficial layer, known as the urogenital triangle, includes the bulbospongiosus, ischiocavernosus, and superficial transverse perineal muscles. The middle layer consists of the perineal membrane and includes deep transverse perineal muscles and the external urethral sphincter. The deepest layer forms the pelvic diaphragm, which is primarily composed of the levator ani (pubococcygeus, iliococcygeus, and puborectalis muscles) and the coccygeus [15]. These muscles interlace with fascia and ligaments that attach to the bony pelvis, creating a dynamic support system.

The fasciae and ligaments of the pelvic floor provide additional structural integrity and are especially significant in female pelvic organ support. These fascial layers transmit mechanical forces and integrate with the muscles to stabilize pelvic contents and support functions like urination, defecation, and sexual response. DeLancey’s model describes three levels of vaginal support based on fascial and ligamentous attachments, all of which are vital in preventing pelvic organ prolapse (POP) [11,13,16].

Physiologically, the PFMs respond to voluntary and involuntary neural stimuli [17]. They contract reflexively during increased intra-abdominal pressure and relax to allow urination, defecation, and vaginal penetration [14]. Their activity is closely integrated with the abdominal muscles, diaphragm, and lower back musculature, forming part of a functional unit responsible for core stability and posture [15]. Electromyography studies reveal that the highest PFM activity occurs in the standing position, supporting the concept that posture directly influences pelvic floor function.

Furthermore, the pelvic floor is richly vascularized by the internal pudendal artery and innervated primarily by the pudendal nerve (S2-S4), with contributions from the autonomic nervous system. Proper neuromuscular function is essential for coordinated contraction and relaxation during various physiological tasks, including micturition, defecation, and sexual activity [15].

Understanding the intricate anatomy and physiology of the female pelvic floor is essential for appreciating how PFMT may improve sexual function and pelvic health. This knowledge underscores the importance of targeted rehabilitation strategies in addressing female sexual dysfunction and pelvic floor disorders.

Female sexual dysfunction: definition and classification 

According to the latest edition of the Diagnostic and Statistical Manual of Mental Disorders (DSM-5), FSD is classified into three major categories: female orgasmic disorder (FOD), female sexual interest/arousal disorder (FSIAD), and genitopelvic pain/penetration disorder (GPPPD) [5].

Women often report a wide range of orgasmic challenges. In line with the views of the International Consultation on Sexual Medicine (ICSM), the International Society for the Study of Women’s Sexual Health (ISSWSH) has acknowledged that current diagnostic frameworks fail to fully capture the spectrum of distressing orgasmic difficulties. FOD is defined by ongoing or recurring disturbances in the frequency, intensity, timing, or pleasurable quality of orgasm that are perceived as distressing and have persisted for at least six months [18,19].

FSIAD refers to a combined lack of sexual desire and difficulty with physiological arousal, such as reduced genital sensations or lubrication, and is influenced by both psychological and hormonal factors. This condition may be lifelong or acquired and is often associated with psychological, relational, or contextual issues [20].

GPPPD encompasses difficulties with vaginal penetration, marked vulvovaginal or pelvic pain during intercourse, fear or anxiety about pain in anticipation of vaginal penetration, and involuntary tightening of the PFMs [19]. This category combines features previously considered separate (dyspareunia and vaginismus) and reflects the interplay of physical, psychological, and relational factors. Understanding these categories is essential for accurate diagnosis, personalized treatment planning, and improving sexual health outcomes in women [20].

Factors contributing to female sexual dysfunction

Biological factors are linked to various forms of FSD, especially those affecting desire and arousal, while their role in orgasm disorders is less clear. Poor general health, chronic conditions, pregnancy, childbirth, and pelvic surgeries like hysterectomy can all negatively affect sexual function [18]. Disorders affecting the cardiovascular, endocrine, neurological, and muscular systems may also contribute to FSD, as can urinary tract symptoms and stress incontinence. Many medications-particularly those for blood pressure, depression, and neurological issues-have been associated with reduced sexual desire, arousal, or orgasm. However, most findings are based on subjective reports rather than controlled studies [20].

Psychological factors play a significant role in FSD, particularly depression and anxiety. Depression is strongly linked to reduced sexual desire, arousal, and orgasm, with evidence suggesting a bidirectional relationship condition potentially worsening the other. Many women report sexual symptoms as part of their depression, although experiences can vary [21]. Anxiety is also associated with FSD, often contributing to conditions like sexual pain and vaginismus [18].

Sociocultural factors such as family values, sexual guilt, and a history of childhood sexual abuse have been linked to female sexual dysfunction, particularly long-term or lifelong issues. Negative past sexual experiences, especially coercion or abuse, are strongly associated with reduced desire and sexual pain in adulthood [22]. The long-term impact often depends on factors like the severity, frequency, and nature of the abuse, as well as the time elapsed since it occurred [23].

Methodology

An extensive review of the literature was carried out across three major scientific databases during the last twenty years: Medline (accessed via PubMed), Web of Science, and Scopus. The search strategy incorporated specific terms such as “pelvic floor muscle training,” combined with related expressions like “pelvic floor muscle exercises,” “female sexual function,” “female sexual dysfunction,” “Kegel exercises,” and “female.” To ensure a thorough investigation, bibliographies of key studies were also reviewed manually for additional relevant sources.

Studies were selected based on inclusion criteria that focused on adult women diagnosed with sexual dysfunction associated with pelvic floor disorders who were undergoing structured PFM interventions. Emphasis was placed on studies that provided comprehensive descriptions of therapeutic protocols. Research designs considered included randomized controlled trials (RCTs), as well as prospective and retrospective cohort studies, limited to English-language publications to ensure interpretative consistency.

As *structured PFM interventions* were defined, those that included a clearly described exercise protocol-specifying the frequency, intensity, duration, and type of pelvic floor muscle exercises (PFMEs). Additionally, the interventions involved supervision or guidance, either individually or in group settings, typically provided by a healthcare professional. Finally, they demonstrated consistency and replicability, meaning the intervention was applied uniformly across participants and could be reliably reproduced in clinical or research contexts. Studies lacking detailed protocol descriptions, unsupervised or informal approaches (e.g., self-directed exercises without clinical guidance), or those not targeting pelvic floor outcomes were not considered structured.

To refine the scope of the review, exclusion criteria were also established. Articles that explored pelvic floor disorders without addressing muscle training components were omitted to retain a focus on muscular rehabilitation. Duplicated records were carefully identified and excluded to uphold data integrity.

In total, 587 references were initially retrieved. Following the removal of 362 duplicates, 227 unique records remained for title and abstract screening. Of these, 191 were excluded based on relevance, leaving 36 articles for full-text assessment. Subsequently, 7 studies were excluded due to the absence of a control group, and 1 study was removed for lacking a clear description of the PFME protocol. Ultimately, 28 studies met the inclusion criteria-comprising 13 interventional and 15 observational designs. The majority of these studies (24 out of 28) were assessed as having moderate methodological quality.

A narrative review approach was selected to allow for a more inclusive and interpretive synthesis of findings. This format enabled the integration of diverse types of evidence, ranging from empirical studies to theoretical analyses and expert opinions. Unlike systematic reviews, which adhere to rigid methodological filters, the narrative format supports a more expansive and adaptive discussion, particularly valuable in exploring multifaceted clinical issues such as those relating to pelvic floor function and its impact on female sexual health.

PFM dysfunction and its association with sexual function

Some researchers suggest that PFM strength may play a role in female sexual function [24]. Weak PFMs have been linked to difficulty achieving orgasm, while stronger muscles, particularly those connected to the clitoris, may enhance arousal and orgasm [25]. Studies have found that women with anorgasmia tend to have weaker PFMs, and higher PFMS has been associated with better sexual function scores. However, not all research agrees, as some studies found no clear link between PFMS and sexual function [26, 27].

Urinary incontinence and sexual function

The relationship between FSD and urinary incontinence (UI), specifically stress urinary incontinence (SUI), appears to be multifaceted, involving both physiological and psychological components. Women with SUI often experience reduced sexual function, which may be due to weakened PFM and hip muscle strength-both of which are crucial for sexual response and continence [26,28]. Embarrassment, fear of leakage during intercourse, and psychological distress associated with incontinence can diminish sexual desire, arousal, and satisfaction. Stronger muscles may help control incontinence and enhance physical ability and confidence during sexual activity, ultimately leading to improved sexual function [28].

POP and sexual function

POP and its relation to sexual function is a complex matter, complexed and influenced by many personal and psychological factors, making it challenging to assess clearly [29]. POP can negatively affect body image and self-confidence, contributing to sexual dysfunction even when physical symptoms are not severe. Improvements in sexual function following POP treatment may often be linked to enhanced body image and reduced emotional distress rather than solely to anatomical correction [29,30].

Postpartum status and sexual function

Postpartum status can significantly impact female sexual function due to physical, hormonal, and psychological changes that occur after childbirth. Vaginal delivery, in particular, significantly increases the risk of pelvic floor disorders such as SUI and POP, primarily due to damage to pelvic support structures, decreased muscle strength, and levator ani muscle injuries [31]. Many women experience some level of sexual dysfunction in the first year after giving birth [32]. PFM training has been shown to improve postpartum sexual function by strengthening these muscles, enhancing blood flow, and supporting genital structures. These exercises can also improve sexual satisfaction and overall quality of life when practiced consistently [33].

Urogynecological conditions and sexual function

FSD is commonly reported among women with urogynecological conditions, including SUI, overactive bladder (OAB), and POP, with symptoms such as embarrassment, coital incontinence, and reduced self-esteem negatively impacting all domains of sexual function. POP, in particular, is associated with high rates of sexual avoidance, dyspareunia, vaginal dryness, and orgasmic issues, especially when combined with incontinence, leading to a cumulative negative effect. Some women may experience worsened symptoms, especially dyspareunia, if both anterior and posterior repairs are performed simultaneously [34].

Approaches and parameters in PFMT

PFMT encompasses various approaches to strengthen the PFMs.

Voluntary Contractions

The most basic form involves conscious contraction and relaxation of the PFMs. These exercises can be performed in different positions (lying, sitting, standing) and with varying contraction durations and repetitions [35].

Biofeedback

This technique uses devices that provide visual or auditory feedback about PFM activity, helping women learn to contract the correct muscles with appropriate intensity. Biofeedback is particularly useful for women who have difficulty identifying or isolating their PFMs [36].

Electrical Stimulation

This involves the application of low-intensity electrical current to the PFMs, causing passive contraction. Electrical stimulation can be beneficial for women who initially cannot contract their PFMs voluntarily [37].

The effectiveness of PFMT is influenced by several key variables. One crucial factor is frequency, with most programs recommending that exercises be performed daily or several times per week to achieve optimal results. Intensity also plays a significant role, referring to the strength of the muscle contractions, which can range from submaximal to maximal effort depending on the individual's capacity and the stage of training [38]. Supervision is another important element; while PFMT can be carried out independently following appropriate instruction, supervised programs-especially those guided by healthcare professionals to produce better outcomes due to enhanced technique and greater adherence. Finally, duration matters both in terms of how long each contraction is held and the total length of the training program. Most PFMT protocols span from six weeks to six months, with continued maintenance exercises recommended to sustain benefits over time [39].

PFMT is widely recommended for managing UI in women, including stress, urgency, and mixed types. An updated Cochrane review analyzed 63 clinical trials involving 4,920 women to evaluate how different PFMT methods-varying in type, frequency, and supervision-affect treatment outcomes, particularly quality of life [40]. The trials assessed various PFMT modalities. Some studies compared traditional isolated pelvic floor exercises with those integrated into broader body movements (coordinated training). Coordinated approaches may provide modest improvements in quality of life compared to direct PFMT alone. When evaluating training frequency, limited evidence suggested that more frequent PFMT sessions might lead to greater improvements, though conclusions were drawn from few, small-scale trials [40]. Comparisons involving the use of resistance devices or different body positions during training lacked sufficient data to determine clear advantages. Delivery of training-whether through individual supervision, group sessions, or digital platforms-was another focus. Individual and group formats showed similar outcomes, while digital methods, such as mobile apps or online instruction, may offer slight benefits over printed materials. However, evidence quality was often rated low due to small sample sizes, inconsistent findings, or inadequate reporting. Adverse events were rare and mostly mild, typically associated with intravaginal or intrarectal training tools. Despite some promising findings, many comparisons lacked robust evidence, highlighting the need for better-designed studies. In conclusion, while PFMT remains a cornerstone of UI treatment, no single training method consistently outperformed others [40].

Based on the systematic review by Jorge et al., the most common protocols used for PFMT in treating female sexual dysfunction share several core features. Typically, these protocols involve voluntary, repeated contractions of the PFMs, often performed both at home and in supervised sessions. The intensity of contractions in most studies was reported as near-maximal or maximal, and the exercises were commonly performed in sets of 8 to 12 repetitions, sustained for 6 to 10 seconds, followed by rest periods of equal duration. Many protocols also included a combination of slow (endurance) and fast (quick flick) contractions, and were performed in various body positions (e.g., lying, sitting, standing). The training duration varied widely - from one month to one year - and supervision ranged from a single instructional session to weekly sessions over several months. Some interventions incorporated biofeedback or electrical stimulation as adjuncts, and over half of the studies included home-based components with adherence monitoring. However, the authors noted that many studies lacked detailed reporting of progression, intensity levels, or adherence strategies, highlighting the need for better-standardized and well-described protocols in future research [41].

Evidence of PFMT effectiveness on female sexual function

Numerous studies have demonstrated the significant impact and crucial role of the PFMs in sexual health, as well as the effectiveness of PFMT in enhancing sexual function across diverse groups of women. The potential benefits and underlying mechanisms through which PFMT contributes to improved sexual health are increasingly recognized.

General population

A retrospective, multicentric cross-sectional study involving women with an average age of 45.76 years - both with and without sexual dysfunction - demonstrated a strong association between PFMS and higher levels of sexual function in women, as reflected by significantly higher Female Sexual Function Index (FSFI) scores. Specifically, the desire, arousal, lubrication, and orgasm domains were markedly improved among women with greater PFM strength, suggesting that stronger pelvic muscles are linked to fewer complaints of sexual dysfunction [42].

**Figure 1:**
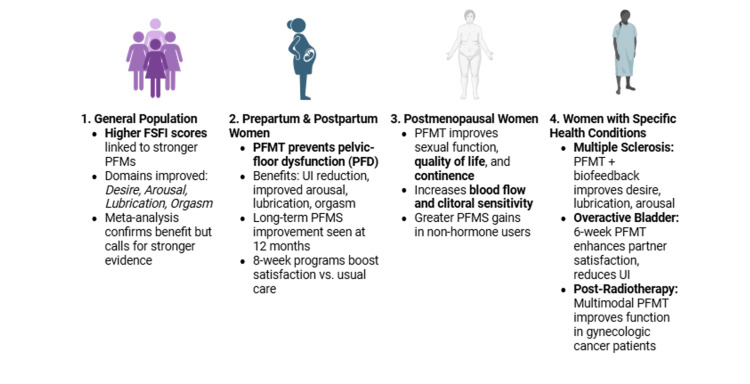
Evidence of the effectiveness of PFMT on female sexual function. Source: [51]. Image credit: Created with BioRender.com (https://BioRender.com/06ifk3o) PFMT, Pelvic floor muscle training

In line with these results, a recent systematic review and meta-analysis further emphasized the positive impact of PFMT on female sexual health. The review concluded that PFMT improves overall FSFI scores, particularly in domains such as arousal, orgasm, and satisfaction. However, it also noted that the certainty of the evidence remains low, indicating the need for more rigorous research in this area (Figure 1) [41].

Prepartum and postpartum women

One group of women, who often experience sexual dysfunction, includes those in the prepartum and postpartum periods. A study demonstrated that most research supports the positive effects of PFMT during these periods in preventing pelvic-floor dysfunction (PFD), particularly by reducing UI symptoms. Improvements have been observed not only in urinary symptoms but also in vaginal laxity, arousal, lubrication, orgasm, dyspareunia, and pain-especially with intensive PFMT protocols. Overall, the evidence supports the use of PFMT as a preventive strategy for PFD in the perinatal period, although further high-quality studies are warranted [43].

Additionally, another study in the same field found that a four-week postpartum PFMT program using the EmbaGYN (Duchesnay Inc., Blainville, Quebec, Canada) and Magic Kegel Master (MagicMotion, West Covina, CA) devices significantly improved pelvic floor strength and reduced symptoms of POP, UI, and fecal incontinence. The Magic Kegel Master device was particularly effective in reducing sexual dysfunction, while the EmbaGYN device proved more effective in addressing UI symptoms [44].

Furthermore, a randomized prospective study investigated the impact of PFMT on postpartum pelvic floor and sexual function in primiparous women. The intervention group, unlike the control group, received weekly 45-minute supervised PFMT sessions for six weeks. Although no significant differences in pelvic floor or sexual function were observed between the groups, after 12 months, the intervention group demonstrated significantly greater PFMS. Both groups experienced notable improvements in pelvic floor and sexual function over time, underscoring the critical role of time in postpartum pelvic floor recovery [45]. 

Finally, a recent randomized controlled trial examined the effects of PFMT on sexual function in postpartum women. Over eight weeks, women who received PFMT showed significantly greater improvements in overall sexual function-particularly in desire, arousal, orgasm, and satisfaction - compared to those receiving usual postpartum care. These findings indicate that PFMT is an effective intervention for enhancing sexual health after childbirth (Figure 1) [46].

Postmenopausal women

Another group that experiences significant benefits from PFMT includes the elderly, particularly postmenopausal women. PFMT has been shown to significantly improve sexual dysfunction in the elderly by enhancing pelvic muscle strength and function, which often decline with age-related hormonal changes. In addition to its positive effects on sexual health, PFMT also contributes to better urinary continence, improved quality of life, and increased physical activity levels. These findings highlight the importance of including PFMT in rehabilitation programs for older adults, not only for managing incontinence but also for addressing the often-overlooked issue of sexual well-being [47]

In addition, a separate study investigating PFMT in 77 postmenopausal women demonstrated its effectiveness in reducing sexual dysfunction in this population. The results showed that PFMT significantly decreased the proportion of women classified as having sexual dysfunction following a 12-week intervention protocol. Beyond its musculoskeletal benefits, PFMT may also enhance sexual function by increasing pelvic blood flow and clitoral sensitivity - mechanisms that are closely associated with improved arousal, lubrication, and orgasm. Although no statistically significant differences were found in FSFI total scores between the intervention and control groups, the clinically meaningful reduction in the prevalence of sexual dysfunction among women who underwent PFMT indicates its therapeutic value. These findings support the implementation of PFMT as a non-invasive, low-risk, and cost-effective strategy to promote sexual health and improve overall quality of life in postmenopausal women [48], while another study highlighted that PFMT increases PFMS more in postmenopausal women who are not using hormone therapy than in women using hormone therapy (Figure 1) [49].

Women with specific health conditions

The benefits of PFMT have also been observed in patients with various diseases or conditions, as well as in those undergoing post-rehabilitation. Particularly, regarding the use of PFMT in the treatment of sexual dysfunction and UI in patients with multiple sclerosis (MS), the available evidence suggests that PFMT- especially when performed under supervision or combined with biofeedback a convenient and effective therapeutic approach. It has been shown to significantly enhance health-related quality of life, with notable improvements in sexual function among female patients with MS [50].

Moreover, the study by Zachariou et al., specifically on sexual dysfunction in women with MS, concludes that PFMT can effectively enhance sexual function and reduce sexual distress in this population. Participants exhibited significant improvements in desire, arousal, vaginal lubrication, and overall sexual satisfaction, as reflected in higher scores on the FSFI and FSDD-R questionnaires. These findings reinforce the role of PFMT not only in strengthening pelvic function but also in alleviating sexual distress, ultimately contributing to an improved quality of life [51].

In addition, the study by Celenay et al. demonstrated that a six-week PFMT program significantly improved sexual function, partner sexual satisfaction, urinary symptoms, and PFMS in women with OAB. Compared to the control group, women who followed the PFMT program experienced significant improvements in FSFI scores across all domains, partner satisfaction, and a reduction in OAB symptoms. These findings support incorporating PFMT into clinical rehabilitation programs for women with OAB [52].

The role of PFMT has also been explored in women who underwent radiotherapy. More specifically, a study examined the effectiveness of physical rehabilitation, including PFMT, in managing sexual dysfunction in women post-radiotherapy. Participants, aged between 20 and 82, received interventions ranging from supervised PFMS and relaxation training combined with manual techniques (e.g., massage) to psychoeducational training and vaginal device use. The study found that a multimodal PFMT intervention was statistically significantly superior to an untreated control group concerning PFMS and sexual function. The effectiveness of a psychoeducational approach was influenced by participants' age and dilation compliance. These findings suggest that rehabilitation approaches incorporating PFMT and vaginal dilator (VD) training may be effective and feasible in improving sexual function in gynecological cancer patients who have undergone radiotherapy (Figure 1) [53].

Clinical implications and practical recommendations 

PFMT is an effective treatment, but its implementation faces challenges. Understanding whether PFMT can be delivered by a range of healthcare professionals, and identifying the barriers, facilitators, and strategies for successful implementation, is essential.

Role of healthcare professionals

Regarding the role of healthcare professionals and the implementation of PFMT for women with POP, a study was conducted within the UK National Health Service (NHS), using various healthcare professional skill mixes. The results showed that PFMT delivery is feasible when carried out by clinicians with an interest in women’s health, provided that adequate training and ongoing support from specialist physiotherapists, multi-disciplinary teams, and management are in place. Increased awareness among staff and management was a key mechanism for change, particularly in areas with a shortage of specialists. In contrast, services with established specialist physiotherapists showed resistance due to role protection and concerns about maintaining quality standards. Staff with prior knowledge of women’s health and strong organizational support felt more confident and comfortable adopting the new role. The results showed that PFMT can be effectively delivered by various healthcare professionals, provided that appropriate training and specialist support are ensured [54].

Another study emphasizes the importance of supervision by healthcare professionals with relevant expertise to ensure that women can correctly contract and relax their PFMs, tailor the exercises to each woman's needs, and avoid discomfort such as back or abdominal pain. It also highlights that motivating women to complete the training program is crucial, as it takes time for the PFMs to strengthen enough to improve symptoms [55].

Adherence and barriers to PFMT

A study that investigated the adherence experience of women with PFD in home-based exercises after an intensive face-to-face physiotherapy treatment aimed to explore their long-term engagement with the exercises. The results showed that adherence depended on the exercise program itself, its perceived efficacy, personal experience with the exercises, intrinsic factors like self-awareness and beliefs, and extrinsic factors such as professional or instrumental feedback. Integrating simple and effective exercises into daily life, enhancing knowledge and awareness of the pelvic floor, and emphasizing the importance of PFM training were found to promote better therapeutic adherence [56].

Moreover, a systematic review and qualitative meta-synthesis investigated the barriers and enablers influencing participation in PFMT among adults of all genders, particularly women. The results showed that opportunities, shaped by social factors, competing demands, and individual capabilities, such as knowledge, understanding, and skill acquisition, were the main influences on PFMT engagement. Clear communication, confidence-building strategies, and the use of technology were suggested to enhance autonomy and participation. Individualized treatment approaches that address social and personal factors are crucial for optimizing self-efficacy and improving adherence to PFMT [57].

In addition, another study investigated the attitudes and barriers toward PFME among women with SUI. The results showed that the main barriers to PFME were a lack of self-discipline, insufficient confidence in correctly performing the exercises, and skepticism about the treatment’s effectiveness based on personal and indirect experiences. Motivators for adherence included achieving desired outcomes, the severity of symptoms, women's expectations, and fear of surgery [58].

The barriers and facilitators to adherence to a prehabilitation program involving PFME and VD, were also investigated, among women undergoing radiotherapy for gynecologic cancer. Facilitators of adherence included high self-motivation, a desire to improve health, recognition of symptom improvement, availability of time, the wish to resume sexual activity, and partner support. Clear instructional materials, effective communication with the physiotherapist, and feedback from the attending physician also encouraged participation. Major barriers included general malaise from cancer treatments, forgetfulness, lack of time, misinformation, lack of coordination with the treatment team, discomfort with the VD, feelings of shame, and the absence of physician feedback [59].

Technological support and patient empowerment

The application of mobile health technology to improve adherence to PFMT among pregnant women was examined through a study focusing on the Kegel Exercise Pregnancy Training (KEPT) app. The findings showed that although pregnant women demonstrated good knowledge about PFMT, their actual practice remained insufficient. Despite the promising features, including reminders, self-monitoring, and a PFMT timer, the study indicated that while the KEPT app may improve usability, further evaluation in a randomized controlled trial is needed to confirm its effectiveness in enhancing PFMT adherence. Pregnant women preferred a straightforward app design and trusted the app more when it included accurate information about PFMT techniques. Future versions of the app may include additional features, such as an antenatal diary, to further engage users and improve adherence [60].

A recent qualitative study investigated the strategies physiotherapists use to empower patients during PFMT. Five themes were identified: building a strong therapeutic alliance, educating to dispel myths and manage expectations, designing personalized PFMT plans, creating a supportive network of professionals and significant others, and offering continuous remote support for self-management. The findings suggest that patient empowerment in PFMT is best achieved through a holistic, patient-centered approach that integrates communication, tailored education, collaboration, and continuous support, providing a useful framework for future interventions and informing empowerment strategies for physiotherapists [61].

Limitations of current literature and future research directions 

Although the effectiveness of PFMT in improving female sexual function is supported by numerous studies, several limitations remain. Some relevant literature may have been missed, potentially affecting the completeness of current evidence. Moreover, there is significant heterogeneity in PFMT protocols and outcome measures, which complicates the comparison of results and limits the ability to draw consistent conclusions. In addition, most studies are short-term in duration, providing limited insight into the long-term sustainability of PFMT benefits. This short follow-up period restricts our understanding of whether the positive effects of PFMT persist over time, especially if the training is discontinued.

Future research should prioritize the use of standardized intervention protocols and include long-term follow-up assessments to improve the reliability of findings and support the integration of PFMT into rehabilitation programs. Studies with extended durations are essential, as improvements may accumulate or change over time. Larger sample sizes are also necessary to increase the statistical power of individual studies and contribute to the development of clinical practice guidelines. Moreover, future research should explore the effectiveness of PFMT both in comparison to, and in combination with, pharmacotherapy, a widely used treatment for sexual dysfunction, and expand to include women with other health conditions or diseases beyond those already studied, to better understand its role in broader clinical populations and ensure the inclusivity and applicability of interventions to real-world settings.

## Conclusions

This narrative review highlights strong evidence supporting the positive impact of PFMT on various aspects of female sexual function, including desire, arousal, lubrication, orgasm, satisfaction, and reduced distress or pain. These benefits are evident in diverse groups, such as postpartum and postmenopausal women, and those with conditions like multiple sclerosis, overactive bladder, or gynecological cancer. The effectiveness of PFMT appears to stem from both physical improvements, such as increased muscle strength, better blood flow, and heightened clitoral sensitivity, and psychological benefits, including enhanced body awareness and reduced anxiety. However, challenges remain in adherence, technique accuracy, and clinician training. Future research should aim to standardize protocols, improve adherence strategies, and include long-term follow-up. Given its safety, cost-effectiveness, and non-invasive nature, PFMT should be integrated into care plans for women with sexual dysfunction, especially when pelvic floor issues are also present. With proper support and implementation, PFMT can significantly improve women’s sexual health and overall quality of life.
